# Recent Progresses in Cancer Nanotherapeutics Design Using Artemisinins as Free Radical Precursors

**DOI:** 10.3389/fchem.2020.00472

**Published:** 2020-06-17

**Authors:** Yalan Wu, Qingping Zeng, Zhiwen Qi, Tao Deng, Fang Liu

**Affiliations:** ^1^Institute of Tropical Medicine and Artemisinin Research Center, Guangzhou University of Chinese Medicine, Guangzhou, China; ^2^Science and Technology Innovation Center, Guangzhou University of Chinese Medicine, Guangzhou, China; ^3^Institute of Chemical Industry of Forest Products, Chinese Academy of Forestry (CAF), Nanjing, China

**Keywords:** artemisinins, anticancer, nanomedicine, chemodynamic therapy, free radicals

## Abstract

Artemisinin and its derivatives (ARTs) are sort of important antimalarials, which exhibit a wide range of biological activities including anticancer effect. To solve the issues regarding poor solubility and limited bioavailability of ARTs, nanoformulation of ARTs has thus emerged as a promising strategy for cancer treatment. A common consideration on nanoARTs design lies on ARTs' delivery and controlled release, where ARTs are commonly regarded as hydrophobic drugs. Based on the mechanism that ARTs' activation relies on ferrous ions (Fe^2+^) or Fe^2+^-bonded complexes, new designs to enhance ARTs' activation have thus attracted great interests for advanced cancer nanotherapy. Among these developments, the design of a nanoparticle that can accelerate ARTs' activation has become the major consideration, where ARTs have been regarded as radical precursors. This review mainly focused on the most recent developments of ARTs nanotherapeutics on the basis of advanced drug activation. The basic principles in those designs will be summarized, and a few excellent cases will be also discussed in detail.

## Introduction

Artemisinin (ART) is derived from the extracts of *Artemisia annua*, a Chinese herbal plant, and it is well-established for the treatment of malaria. Up to date, several semi-synthetic derivatives of ART have been synthesized and clinically applied, including artesunate (AS), dihydroartemisinin (DHA), artemether (ARM), and arteether (ARE). Besides anti-malarial usage, the therapeutic effect of ARTs has also been extended to non-parasitic diseases, such as inflammatory disease and cancer (Krishna et al., 2008; Li, 2012). ARTs have been proved to be effective to treat a wide range of cancers both *in vitro* and *in vivo* (Wong et al., 2017). Although the anticancer mechanism of ARTs is not fully discovered yet, some investigations have pointed out that Fe^2+^ may be the natural catalyst in cells that mediates ARTs' activation. Fe^2+^-catalyzed opening of the endoperoxide bridge is through an Fenton-like reaction, which generates alkyl radicals and reactive oxygen species (ROS) that can subsequently react with susceptible protein targets (O'neill et al., 2010; Wang et al., 2015; Deng et al., 2020). Tumor cells often have elevated iron accumulation, which has been regarded as one of the leading reasons why ARTs can kill cancer cells selectively (Pfeifhofer-Obermair et al., 2018; Zhang et al., 2018).

However, using ARTs for real cancer therapy has been limited by their poor solubility, low bioavailability, and extremely short half-life *in vivo* (De Vries and Dien, 1996; Dwivedi et al., 2015). Nanotechnology offers the possibilities to overcome those drawbacks, thus being extensively used in the delivery of drugs especially for those with difficulty in water solubility (Kalepu and Nekkanti, 2015; Patra et al., 2018). A general method for ARTs delivery is loading the drug molecules within a certain nanocarrier, which could be made from inorganic or organic substrates, or both of them (Wang et al., 2016b; Mangrio et al., 2017; Manjili et al., 2018; Xiao et al., 2020). Additional chemistry for achieving tumor targeting and controllable drug release is often considered during the design (Tran et al., 2016; Jabbarzadegan et al., 2017; Akbarian et al., 2020). Up to date, numerous nanosystems have been reported for ARTs delivery and controlled release, and those studies have been well-summarized in previous review articles (Chen et al., 2009; Aderibigbe, 2017; Charlie-Silva et al., 2018). Another much advanced strategy to improve the therapeutic effect of ARTs is the method of so-called chemodynamic therapy (CDT), which has been attracting interests for cancer treatment via localized Fenton reaction (Tang et al., 2019; Zhao et al., 2019). Therefore, the ways of the delivery and controlled release of active Fenton reagent such as Fe^2+^ to accelerate ARTs' activation are attracting the great attentions in the design of ART-CDT nanotherapeutics. The main target of those nanosystems is to cause free radical burst thus leading to cell toxicity, whereby ARTs are usually used as radical precursors. In the present review, we will focus on the recent progress mostly within the last 5 years in cancer nanotherapeutics design using ARTs as free radical precursors. The discussion will be divided into four parts according to the differences of technique aspects.

## ARTs Nanotherapeutics

### Iron Oxide-Based ARTs Nanotherapeutics

With the common sense that Fe^2+^ ions are the most active species that can activate ARTs, iron oxide-based inorganic and inorganic/organic hybrid nanoparticles have therefore been considered for ARTs' delivery and controlled activation. Most of these nanoparticles are made from the mixtures of Fe^2+^ and Fe^3+^, which are potentially able to release active Fe^2+^ in mild acidic environment of tumor tissues and acidic intracellular compartments. In addition, many oxide nanoparticles are paramagnetic and serve as excellent contrast reagents for magnetic resonance imaging (MRI), which offers the possibilities for the development of advanced theranostics (Bao et al., 2018). Up to now, several iron oxide–based ART nanotherapeutics have been reported and proved to be effective *in vitro* and *in vivo* in cancer therapies. For example, Chen et al. have reported a nanoparticle Fe_3_O_4_@C/Ag@mSiO_2_ with mesoporous properties, which can load with ART up to 484 mg/g. This nanoparticle was internalized by cancer cells mainly through endocytosis pathways, Fe_3_O_4_ in acidic endo/lysosome was able to release catalytic Fe^2+^ that further activated the generation of radical species from ART. The authors found elevated apoptosis to HeLa cells treated with this nanoparticle compared with ART itself (Chen et al., 2014). By following this design, the same group has presented another Fe_3_O_4_-based nanosystem Fe_3_O_4_@C@MIL-100(Fe), where dihydroartemisinin (DHA) was encapsulated as radical precursors. More interestingly, this system is able to increase the accumulation of DHA and Fe^2+^ within targeted cancer cells under the guidance of an external magnetic field. Much improved therapeutic effect has been found *in vivo* in this study (Wang et al., 2016a).

The cases above have revealed that targeting to the acidic endo/lysosome is a promising way to achieve Fe^2+^/Fe^3+^ release from iron oxide nanoparticles. By following these studies, several ARTs nanosystems have been reported most recently (Wang D. et al., 2017; Zhang et al., 2017; Pan et al., 2018; Guo et al., 2019; Li et al., 2019; Qin et al., 2019). Generally, nanoparticles are easy to be chemically modified with additional ligands to strengthen the functionalities. For instance, Zhang et al. (2016) reported a multi-functional nanoparticle, in which hyaluronic acid (HA) was grafted on the ART doped mesoporous Fe_3_O_4_ core structures. Controlled release of Fe^2+^ and ART is thus achievable since tumor cells are often enriched with hyaluronidases (Mcatee et al., 2014). Such an HA-gate method has also been applied in another report by the same research group (Zhang et al., 2017). In a recent report, an additional cancer cell-targeting and membrane-penetrating RGD peptide was integrated on a Fe_3_O_4_@cisplatin/ART nanoparticle to form ART-loaded cRGD-AFePt@NPs for cancer targeted co-delivery of cisplatin and ART, and controlled drug activation (Gao et al., 2018). The most recently reported iron oxide nanosystems for ARTs-based cancer therapeutics have been summarized and listed in Table 1. We do believe that this sort of design will be continually applied in the fabrication of advanced ARTs nanodrugs in the future.

**Table 1:** Recent developments of iron oxide nanoparticle-based ARTs nanotherapeutics.

	**Nanotherapeutics**	**ARTs**	**DLC**	**Activator**	**Strategy**	**Evidence**	**References**
1	Fe_3_O_4_@C/Ag@mSiO_2_	ART	48.4%	Fe^2+^/Fe^3+^	ART-CDT	*in vitro*	Chen et al., 2014
2	Fe_3_O_4_@C@MIL-100(Fe)	DHA	80.4%	Fe^2+^/Fe^3+^	ART-CDT	*in vitro/in vivo*	Wang et al., 2016a
3	Mn_3_[Co(CN)_6_]_2_@MIL-100 (Fe)	AS	53.1%	Fe^2+^/Fe^3+^	ART-CDT	*in vitro/in vivo*	Wang D. et al., 2017
4	ART@HQZFNPs	ART	23.0%	Fe^2+^/Fe^3+^	ART-CDT	*in vitro*	Pan et al., 2018
5	MNP-ART, MNP-DHA, MNP-AS	ART, DHA, AS	15.3%, 15.3%, 15.7%	Fe^2+^/Fe^3+^	ART-CDT	*in vitro*	Guo et al., 2019
6	DHA-MLPs	DHA	82.1%	Fe^2+^/Fe^3+^	ART-CDT	*in vitro/in vivo*	Li et al., 2019
7	Fe_3_O_4_@SiO_2_-ART-HNPa	ART	45.2%	Fe^2+^/Fe^3+^	ART-CDT, PDT	*in vitro*	Qin et al., 2019
8	A-TiO_2_-IONPs/ART	ART	27.5%	Fe^2+^/Fe^3+^	ART-CDT, PDT	*in vitro/in vivo*	Zhang et al., 2017
9	HA-mFe_3_O_4_/ART	ART	52.8%	Fe^2+^/Fe^3+^	ART-CDT, AMF	*in vitro/in vivo*	Zhang et al., 2016
10	cRGD-AFePt@NPs	ART	5.2%	Fe^2+^/Fe^3+^	ART-CDT Cisplatin	*in vitro*	Gao et al., 2018
11	Fe_3_O_4_@MnSiO_3_-FA	ART	21.9%	Mn^2+^, Fe^2+^/Fe^3+^	ART-CDT	*in vitro/in vivo*	Chen et al., 2015
12	ART-MSP loaded with ICG	ART	22.5%	Fe^2+^/Fe^3+^	ART-CDT, PTT	*in vitro/in vivo*	Ding et al., 2018

*DLC, drug loading capacity; MNP, magnetic nanoparticle; MSP, porous magnetite supraparticles; AFM, alternating magnetic field (AMF) irradiation; ZFNPs, Zinc ferrite (ZnFe_2_O_4_) nanoparticles; HQ, 8-hydroxyquinoline; MLPs, magnetic nano-liposomes; HA, hyaluronic acid; ICG, indocyanine green; HNPa, 3-[1-hydroxyethyl]-3-devinyl-131-b,b-dicyanomethylene-131-deoxopyropheophorbide-a*.

### Iron-Containing Soft Matters for ARTs Delivery and Activation

Polymeric soft matters including natural proteins, polysaccharides, and synthetic polymers have gained great attentions for the fabrication of nanotherapeutics. Numerous small molecular drugs have been provided as nanoformulations in clinic applications by using polymeric soft matters as drugs' nanocarriers (Malmsten, 2006; Liechty et al., 2010). Different to other small molecular drugs, a successful delivery system for ARTs-based cancer treatment requires not only effective drug release but also efficient peroxide bridge activation. Therefore, the soft matters that can serve as the carriers for both ARTs and the catalyst Fe^2+^/Fe^3+^ are much extensively studied.

**Figure 1:**
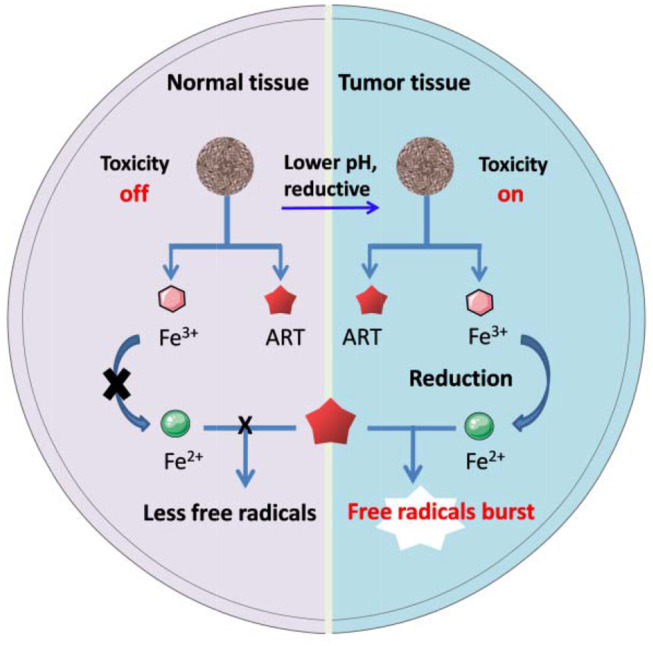
Schematic artwork of tumor selective ART-CDT through iron-containing polymer nanoparticles.

Transferrin, a kind of iron containing proteins, has been used as nanocarriers to directly deliver ARTs for cancer therapy in several cases. One of the earliest reports presented a drug-protein complex by covalently conjugating an ART derivative with the carbohydrate chains of transferrin. *In vivo* evidence showed that linkage with transferrin can greatly enhance the therapeutic effect of ART on the mice implanted with human ovarian cancer cells, HO-8910 (Nakase et al., 2008). It has also been found that transferrin with ART tagged with lysine residues was less effective than those with ART tagged to the carbohydrate chains (Lai et al., 2005). Direct conjugation of ARTs to transferrin may lead to some drawbacks such as limited loading efficiency and potential suppressive effect to the intrinsic biological functions of transferrin. To overcome the drawbacks, some much advanced nanosystems have been presented (Zhang et al., 2015a,b; Ji et al., 2019; Luo et al., 2019). For example, in a recent study, Xing's group reported a system for pH-triggered cancer chemotherapy by coating graphene oxide (GO) with both transferrin and DHA, where transferrin served as not only a cancer cell-targeting ligand but also an iron carrier (Liu et al., 2015). Most recently, Tian's group has presented a DHA encapsulated liposomal nanosystem functionalized with transferrin as tumor-targeting ligands. Elevated oxidative stress was observed specifically at the tumor site and enhanced tumor eradication was achieved by their nanosystem (Yu et al., 2020). Methemoglobin (MHb) is the most common iron-containing protein, which has also been utilized in ARTs' delivery and controlled activation through the similar mechanisms to transferring (Li et al., 2017). The iron ion in both transferrin and MHb exists mainly as Fe^3+^, it will convert to Fe^2+^ under reductive intracellular microenvironment, thus mediating ARTs' activation (Liu et al., 2015). Other ARTs nanosystems based on Fe^3+^ bonded polymers may follow a similar activation mechanism as stated in Figure 1.

Polydopamine (PDA) is made from the polymerization of natural dopamine monomers, which shows the abilities to tightly coordinate with Fe^2+^ and Fe^3+^ ions. The release of these ions from polydopamine depends on the degradation of polymer structure as well as the pH values surrounding it. Generally, lower pH often leads to greater release. A recent study presented a hollow nanosphere DHA@HPDA-Fe, in which polydopamine vesicles were loaded with DHA and Fe^3+^. *In vivo* evidence showed that the anticancer efficacy of DHA@HPDA-Fe was 3.05 times higher compared with free DHA (Dong et al., 2019). Besides, some other biocompatible polyphenolic compounds such as tannic acid have similar binding and releasing futures toward Fe^2+^ and Fe^3+^(Kell, 2009; Du et al., 2019; He et al., 2019). These polymers could also be considered for construction of ART's nanotherapeutics, but the related research is still rare.

### ARTs Nanotherapeutics Activated by Other Metal Ions

In addition to Fe^2+^, some other transition metal ions such as Zn^2+^, Mn^2+^, Ni^2+^, Ti^3+^, Cu^2+^, and Co^2+^ ions could also be used as catalysts to catalyze Fenton reaction (Bokare and Choi, 2014). This may greatly expand the alternatives when considering a catalyst for ARTs' activation. For instance, Chen et al. have reported a Mn^2+^-doped nanoparticle Fe_3_O_4_@MnSiO_3_-FA for successful delivery of AS to tumors in mice models. It was found that both Fe^2+^ and Mn^2+^ would contribute to the catalyzed activation of AS. The authors further suggested that Mn^2+^ would be more effective to promote Fenton-like reaction and lead to more free radicals generated from AS (Chen et al., 2015). Zhou et al. presented a multifunctional nanoparticle ART-loaded mesoporous NiO (mNiO) for cancer cell targeted therapy, in which Ni^2+^ served as the catalyst for ART's activation. The authors found the evidence that mNiO can undergo degradation in acidic tumor microenvironment and release free Ni^2+^, which benefits the tumor selectivity over normal tissues (Liu et al., 2018). Although Mn^2+^ and Ni^2+^ doped with ARTs nanocomposites have exhibited catalytic effect in ARTs' activation, and there are still many ions alternative to choose for testing, the biocompatibility issues of such nanosystems should be greatly concerned for the purpose of real use.

### Nanotherapeutics With the Combination of ART-CDT and Other Therapeutic Techniques

ARTs with peroxide bridges offer excellent radical precursors for the design of ARTs-based chemodynamic therapy (ART-CDT), which often involves the dynamic activation of ARTs and the generation of toxic free radicals (Tang et al., 2019). H_2_O_2_, hydroxyl radicals, and alkyl radicals are considered as the major radical species from the consequences following ART activation (Deng et al., 2019). Another radical-based therapeutic strategy is photodynamic therapy (PDT), whereby photo-mediated generation of singlet oxygen (^1^O_2_) is the major scientific bases for therapy. ART-CDT and PDT are all ROS involved, and the combination therapy based on both is thus attracting increasing interests (Feng et al., 2018).

More recently, Jin et al. have developed a nanosystem Fe_3_O_4_@SiO_2_-ART-HNPa for cancer therapy with the combination of ART-CDT and PDT. Within their core-shell nanoparticle Fe_3_O_4_@SiO_2_, ART and a porphyrin photosensitizer HNPa were encapsulated into the mesoporous structure of SiO_2_. In this system, ART was considered to generate ROS and alkyl radicals through pH-mediated Fenton-like reactions. Meanwhile, ^1^O_2_ generation from HNPa and molecular oxygen was mediated by NIR light (700 nm) excitation. Enhanced anticancer effect has been observed compared with PDT or the Fenton-like reaction alone (Qin et al., 2019). Recently, a nanoscale metal organic framework (nanoMOF) doped with Fe^3+^, ART, and a photosensitizer have been constructed by Tang's group for synergistic cancer therapy based on ART-CDT and PDT. In this study, Fe^3+^ and the photosensitizer 4,4,4,4- (Porphine-5,10,15,20-tetrayl)tetrakis(benzoic acid) (TCPP) were coordinated to form the nanoMOF core structures followed by the encapsulation of DHA. To prevent non-specific DHA release, the nanoMOF was further coated with a layer of CaCO_3_. A 655 nm laser was applied for PDT during the combination therapy. Both the *in vitro* and *in vivo* results indicated synergistic therapy effect from this nanosystem, which could ablate the tumor completely on the breast cancer cell 4T1-bearing mice (Wan et al., 2019).

Photothermal therapy (PTT) is a therapeutic approach of heat ablation, which uses the heat converted from pathologic tissue localized light irradiation (Vines et al., 2019). It has been found that PTT is able to improve Fenton-reaction-based therapy through enhancing the generation of ROS (Hu et al., 2017; Chen et al., 2020), thus holding the great promise to be combined with ART-CDT in cancer treatment (Wang et al., 2016b; Hu et al., 2017; Ding et al., 2018; Liu et al., 2018). Most recently, Wang et al. have presented a dual functional magnetic nanoparticle MSP@ART@P for cancer treatment based on the combination of ART-CDT and NIR laser (785 nm) mediated PTT. In their design, ART was firstly loaded into a magnetic iron oxide nanoparticle. A poly (aspartic acid) polymer linked with dopamine and indocyanine green (ICG) was then coated with the ART-loaded core nanoparticle. The authors have experimentally confirmed the cancer ablation effect from ART-CDT, and that the therapeutic effect could be further promoted by photothermal effect. This research offers a way to elevate cancer tissue–localized ROS generation through photosensitizer/O_2_-independent manners (Ding et al., 2018).

## Summary and Perspectives

The peroxide bridges existing in ARTs make them the most attractive free radical precursors for radical-based CDT. Fe^2+^ and Fe^2+^-containing complexes such as heme are considered as the major catalysts that can activate ARTs within cells through Fenton-like reactions. Based on these considerations, some iron oxide nanoparticle-based ARTs delivery and activation systems have been developed as discussed above. The activation relies on Fe^2+^ generation either from the direct ion release of iron oxide nanoparticles or reductive conversion of Fe^3+^. The controlled release and activation of ARTs could also be achieved by loading ARTs with iron-containing soft matters, which include natural iron-containing proteins such as transferrin and hemoglobin, as well as polyphenols chelated with Fe^2+^/Fe^3+^. The recent developments in this field have been summarized, and a few excellent cases have been discussed in details. Besides Fe^2+^, cancer cell-targeted release of Ni^2+^ and Mn^2+^ has also been proven effective in ARTs' activation, resulting in high antitumor activity *in vitro* and *in vivo*. In principle, some other metal ions that catalyze Fenton-like reaction can also serve as the activators for ART-CDT. However, in such kinds of design, the potential toxic issues due to the overloading of metal ions have to be carefully considered. An alternative way against the delivery of toxic metal ions is to enhance the catalytic effect of intrinsic Fe^2+^ by delivering a proper iron chelate into the cells. For example, heme, a protoporphyrin IX-Fe^2+^ complex, has been proven to be more effective in catalyzing activation of ARTs. The way to improve the formation of intracellular heme would be a possible strategy to enhance the anticancer activities of ARTs, which have gained evidences in some recent studies (Wang J. et al., 2017; Chen et al., 2019).

Combination of ART-CDT and other advanced therapeutic methods will be the most promising trend in the design of ART-based nanotherapeutics. ART-CDT combined with PDT or PTT has shown synergistic effect for cancer therapy. Elevated ROS generation from ARTs is one of the major causes of synergistic effect in these combination therapies. Ultrasound irradiation mediated sonodynamic therapy (SDT) has emerged recently as a new method for cancer therapy (Canavese et al., 2018). Compared to other external stimuli such as light, ultrasound irradiation has better tissue penetrating capability. More recently, several studies have confirmed the possibilities to generate ROS in aqueous mediums through ultrasound irradiation (Gong et al., 2015; Giuntini et al., 2018). Similar to PPT, SDT is also able to release heat locally, thus making the potentials to accelerate the Fenton-like reaction on ARTs. The combination of ART-CDT and SDT, therefore, will be a promising option in the design of advanced ART nanotherapeutics in the future.

## Author Contributions

YW, ZQ, and TD drafted this manuscript. FL and QZ revised it. All authors contributed to the article and approved the submitted version.

## Conflict of Interest

The authors declare that the research was conducted in the absence of any commercial or financial relationships that could be construed as a potential conflict of interest.
